# LC‒MS/MS and transcriptome analyses reveal saliva components of the seed-feeding truebug *Pyrrhocoris apterus*

**DOI:** 10.1007/s44297-023-00021-w

**Published:** 2023-12-15

**Authors:** Qian Lin, Hui-Jie Wu, Zhuo-Qi Liu, Yi Wan, Hai-Jun Xu, Jin-Li Zhang

**Affiliations:** State Key Laboratory of Rice Biology and Breeding, Key Laboratory of Biology of Crop Pathogens and Insects of Zhejiang Province, Institute of Insect Sciences, College of Agriculture and Biotechnology, Zhejiang University, 866 Yu-Hang-Tang Ave, Hangzhou, 310058 China

**Keywords:** *Pyrrhocoris apterus*, Hemiptera, Saliva, LC‒MS/MS, Transcriptome, RNAi

## Abstract

Saliva secretion is essential for successful feeding by piercing–sucking insects. Despite extensive studies of the saliva composition of phloem- and blood-feeding insects, less is known about the oral secretions of seed-feeding insects. The firebug *Pyrrhocoris apterus* is a polyphagous insect that feeds primarily on dry seeds of various plant species. Here, liquid chromatography‒mass spectrometry/mass spectrometry (LC‒MS/MS) was used to identify 165 *P. apterus* salivary proteins, including 110 gelling-specific proteins, 24 watery-specific proteins, and 31 proteins common to both groups. Transcriptomic analysis of saliva glands identified 567 potential salivary proteins, of which 87 overlapped with those detected by LC‒MS/MS. Comparative analysis showed that 98 out of 165 (59.4%) *P. apterus* saliva proteins were associated with extra-oral digestion and detoxification and had homologs in ten other species of arthropods examined. In addition, 67 proteins were specific to *P. apterus*, of which 22 proteins were defined with unknown functions. RNA interference-mediated gene silencing assays indicated that *P. apterus*-specific proteins were vital for *P. apterus* survival. Our findings shed light on the function of salivary proteins in feeding by seed-feeding insects and the evolution of feeding habits in piercing–sucking insects.

**Supplementary Information:**

The online version contains supplementary material available at 10.1007/s44297-023-00021-w.

## Introduction

Hemipteran insects have delicate piercing–sucking mouthparts with a stylet that is highly specialized for penetrating tissues and sucking out liquids [1–4]. Many phytophagous hemipterans, such as aphids, planthoppers, and true bugs, insert their stylets at the feeding site and produce gelling or watery saliva at different stages of the feeding process [1, 5]. Gelling saliva is thought to be produced by the principal salivary glands and secreted onto the surface of food [6–8]. As the stylet advances through the plant tissue, gelling saliva is secreted incrementally and forms a continuous solid sheath (salivary flange), which encases the style and provides mechanical stability and lubrication [8–10]. In addition to acting as a salivary sheath, gelling saliva contains digestive enzymes that minimize mechanical injury to plant tissues [5, 11]. After stylet penetration, a large amount of watery saliva is secreted into plant tissues, which contain bioactive components involved in the suppression or induction of plant defense responses [2, 8, 12, 13]. With the help of a flow of saliva, phytophagous hemipterans suck out liquid nutrients from plants while simultaneously transmitting various plant diseases. Given that saliva is involved from the start of this insect–plant encounter, identifying its components is the first and essential step for understanding the biological function of insect saliva.

Recently, numerous salivary proteins produced by phloem-feeding insects have been identified, and their secretion is crucial for safe and successful feeding [2, 9, 12, 14–19]. For instance, the salivary proteins Mp56, Mp57, and Mp58 secreted by the green peach aphid *Myzus persicae* inhibit insect fecundity by activating the plant defense response, whereas MpC002 and Mp2 are essential for successful feeding and reproduction by these aphids [12, 20–22]. In the brown planthopper *Nilaparvata lugens*, a notorious sap-sucking insect pest on rice in East Asia, the salivary proteins Nlshp and Nlsalivary protein-3 are indispensable for the formation of the salivary sheath and rice feeding [9, 23]. In addition, various enzymes and bioactive proteins, such as oxidoreductases, hydrolases, peptidases, proteases, isomerases, transferases, mucin, vitellogenin, and calmodulin, have also been identified in the saliva of phytophagous sap-sucking insects, and contribute significantly to insect–plant interactions [6, 17, 19, 24–28]. Despite the functional importance of salivary proteins identified in phloem-feeding insects, the salivary components of piercing–sucking insects that feed on seeds remain elusive.

The firebug, *Pyrrhocoris apterus* (Heteroptera: Pyrrhocoridae), is a gregarious species that has been used in a variety of morphological, developmental, ecological, genetic, and evolutionary studies [29]. As a polyphagous insect, *P. apterus* primarily feeds on dry seeds of various plant species, including those belonging to the Malvaceae, Rosaceae, and Tiliaceae [30–32]. Additionally, it occasionally sucks plant sap, water, and dead animal matter [31]. Over the past two decades, *P. apterus* has expanded its distribution rapidly from Eurasia to other areas of the world, including North America and south-eastern Australia, due, in part, to the warming climate and human activity [29, 33–35]. Thus far, the saliva components of *P. apterus* have not yet been identified, which limits our understanding of the feeding process of this seed-feeding insect as well as its habitat expansion.

In this study, we determined the proteinaceous components of the watery and gelling saliva of *P. apterus* using liquid chromatography-tandem spectrometry (LC‒MS/MS) and transcriptomic analysis. Our results indicated that watery and gelling saliva of *P. apterus* were enriched with digestive enzymes and oxidoreductases essential for extra-oral digestion. RNA interference (RNAi)-mediated gene functional assays indicated that species-specific salivary proteins may be vital for *P. apterus* survival. These findings further our understanding of the evolution of feeding habits in piercing-sucking insects.

## Materials and methods

### Insect preparation

The firebug *P. apterus* was originally collected in Urumqi, Xinjiang Uygur Autonomous Region, China, in 2020. The insects were maintained in a walk-in chamber at 26 °C under a photoperiod of 16 h light/8 h dark, and were supplied with water and red clover seeds (*Trifolium pratense* L). *P. apterus* exists in both long- and short-winged morphs. A short-wing (SW) population was used in this study, in which long-winged individuals are scarce (< 1%).

### Collection of watery saliva proteins

**Fig. 1:**
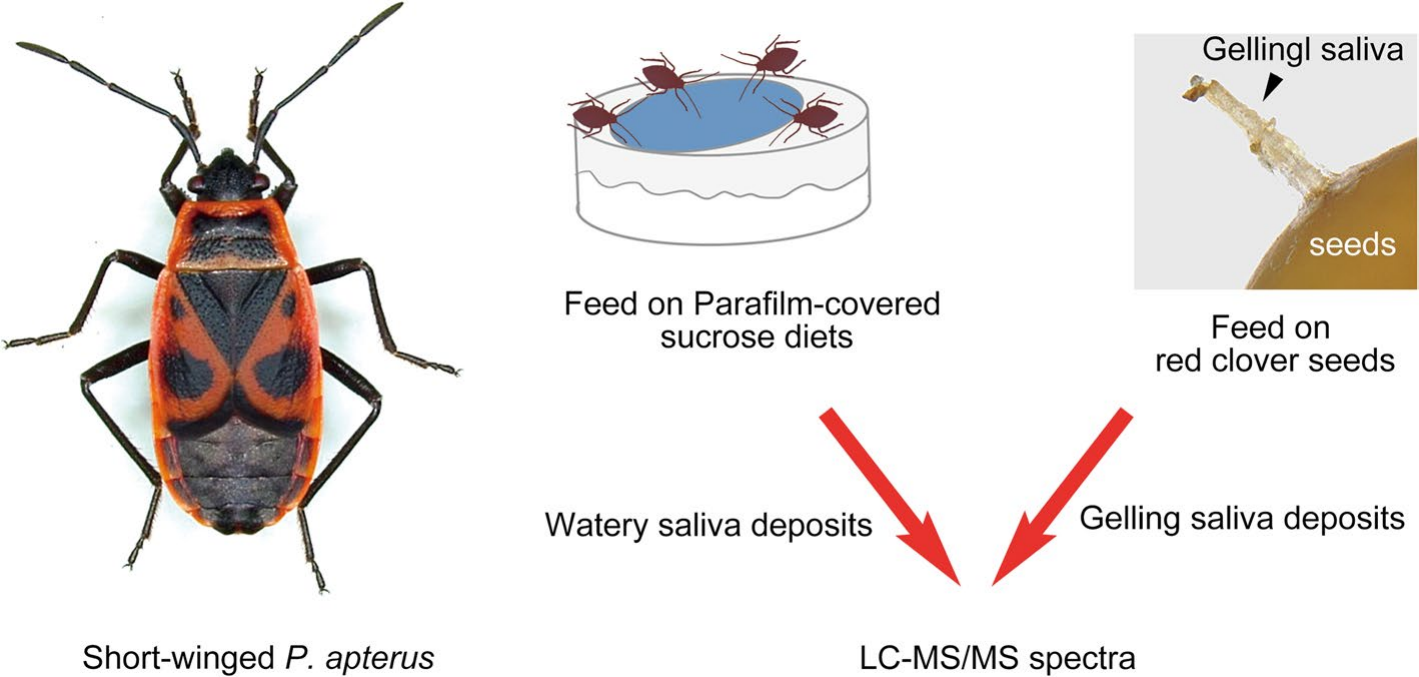
Overview of workflow used to identify salivary proteins. To collect watery salivary, *P. apterus* was allowed to feed on an artificial diet (1.5% sucrose solution), and the diet fluid was concentrated for LC‒MS/MS analysis. To collect gelling saliva, insects were allowed to feed on red clover seeds and salivary sheaths were collected for LC‒MS/MS analysis

A 1.5% sucrose diet solution was prepared by dissolving sucrose into Milli-Q ultrapure water and filtering it through 0.22-µm filters. To perform artificial feeding, 1 mL sucrose solution (1.5%) was sandwiched between two layers of stretched Parafilm and placed on top of a 35-mm Petri dish with the exposed diet facing upward (Fig. 1). Approximately 1,000 adult *P. apterus* were allowed to feed on each dish for 24 h. The diet solution containing secreted saliva was pooled and then centrifuged at 7,*000* × *g* for 30 min at 4 °C. Then, the supernatants were ultrafiltered using a 3-kDa molecular-weight cut-off Amicon Ultra-15 Centrifugal Filter Device (Millipore). Subsequently, the saliva solution was concentrated to ~ 25 μL using a freeze-dryer (Alpha 1–2 LD plus, Martin Christ).

### Collection of gelling saliva proteins

To collect gelling saliva, ~ 250 *P. apterus* adults were allowed to feed on red clover seeds for 24 h in a plastic chamber (Fig. 1). The salivary sheaths were carefully removed from the surface of seeds using forceps under a stereomicroscope (Leica S8AP0). Subsequently, the salivary sheaths were pooled and washed in lysis buffer (4% CHAPS, 2% SDS, and 2% DTT), followed by grounding with liquid nitrogen. The sheath powder was then heat-solubilized in 40 μL lysis buffer for 10 min. Last, the saliva solution was denatured in 9 M urea at room temperature for 1 h, and then concentrated to a volume of ~ 30 μL using a freeze-dryer.

### Polyacrylamide gel electrophoresis

The collected watery and gelling saliva solutions were mixed in a ratio of 1:5 protein loading buffer (12% SDS, 300 mM Tris–HCl, 600 mM DTT, 60% glycerol, and 0.6% bromophenol blue), and then denatured by boiling for 5 min. Then, salivary proteins were separated by sodium dodecyl sulfate‒polyacrylamide gel electrophoresis with a 6% stacking and 12% separating gel. Protein bands were visualized under staining with Coomassie brilliant blue. Subsequently, the protein bands exhibiting a blue color were excised for LC‒MS/MS analysis.

### LC‒MS/MS analysis

The excised SDS‒PAGE gels were decolorized in de-staining buffer [50% acetonitrile (ACN), 50 mM triethylammonium bicarbonate (TEAB)], followed by dehydration in 100% ACN. The gels were treated with 1 ml DTT (10 mM) for 40 min at 56 °C and then alkylated with 1 mL iodoacetamide (50 mM) for 30 min in the dark. Subsequently, the gels were washed with destaining buffer and treated with 100% ACN one more time. The decolorized gels were resolved in a 100 μL digestion solution (0.01 mmol TEAB, 1 μg trypsin, and 0.3 μg CaCl2) overnight at 37 °C. Then, peptides were extracted from the supernatant using 0.1% formic acid. The peptide mixture was loaded onto the trap column of the EASY-nLCTM 1200 UHPLC system (Thermo Fisher Scientific) coupled with a Q ExactiveTM HF-X mass spectrometer (Thermo Fisher Scientific). The LC‒MS/MS proteomics data were deposited to the ProteomeXchange Consortium (GenBank project accession: PXD040558).

### Bioinformatics analysis

Protein identification was performed using Proteome Discoverer v2.2 (Thermo Fisher Scientific) against the *P*. *apterus* genomic database (32,085 entries). The following search parameters were used: (*i*) type of quantification, precursor quantification; (*ii*) fragment mass tolerance, 0.02 Da; (*iii*) enzyme, trypsin; (*iv*) max missed cleavage sites, 2; (*v*) static modification, carbamidomethyl (C); (*vi*) precursor mass tolerance, 10 ppm (M); (*vii*) dynamic modification, oxidation (M); and (*viii*) N-terminal modification, acetyl (N-terminal). Peptide Spectrum Matches (PSMs) with a confidence level > 99% and proteins with at least one unique peptide were considered as confidence. In addition, peptides with false discovery rates (FDRs) < 0.05 were considered for identification. The presence of signal peptides was predicted using the SignalP Server (v5.0) (https://services.healthtech.dtu.dk/services/SignalP-5.0/). The prediction of transmembrane helices in proteins was predicted using TMHMM-2.0 (https://services.healthtech.dtu.dk/services/TMHMM-2.0/).

### Spatiotemporal expression of salivary genes

To investigate the temporal expression of target genes, total RNAs were isolated from eggs (*n* = 100), first-instar (*n* = 50), second-instar (*n* = 20), third-instar (*n* = 20), fourth-instar (*n* = 10), fifth-instar nymphs (*n* = 10), and adult females (*n* = 10) and males (*n* = 10) using RNAiso Plus (Takara) according to the manufacturer’s instructions. To investigate the spatial expression of target genes, the abdominal cuticle, fat body, ovary, testis, head, gut, and salivary gland were dissected from each adult (*n* = 20) and used for RNA isolation. A total of 1 µg RNA per sample was used to construct a sequencing library using a NEBNext Ultra RNA library prep kit for Illumina (NEB) according to the manufacturer’s instructions. Library fragments 250–300 bp in length were purified using an AMPure XP system (Beckman Coulter). Clustering of the index-coded samples was performed on a cBot Cluster generation system using a TruSeq PE cluster kit v3-cBot-HS (Illumina). cDNA libraries were sequenced on an Illumina NovaSeq 6000 platform, and 150 bp paired-end reads were generated (GenBank project accession: PRJNA954282).

### Mapping and gene expression analysis

After RNA sequencing (RNA-seq), clean reads were generated from raw data after removing adapter, poly-N, and low-quality reads using the fastp algorithm (v0.12.4) [36]. The clean reads were mapped against the *P. apterus* draft genome using hisat2 (v2.1.0) [37], and the transcript abundance was quantified using StringTie (v1.3.5) [38]. Fragments Per Kilobase of transcript per million mapped reads (FPKM) was used to quantify the expression level of each transcript. To generate heatmaps, FPKM of each gene was *z*-score transformed and clustered using the online OmicShare tool (www.omicshare.com/tools/Home/Soft/heatmap). Gene Ontology (GO) enrichment analysis of genes was performed using the online OmicShare tool (www.omicshare.com/tools/home/report/goenrich.html).

### Comparative analysis of insect saliva

We compared *P. apterus* saliva proteins with those from ten published arthropod species: five aphids [*Acyrthosiphon pisum* [39], *Macrosiphum euphorbiae* [40], *M. persicae* [41], *Diuraphis noxia* [42], and *Rhopalosiphum padi* [41], two true bugs [*Halyomorpha halys* [19, 43] and *Riptortus pedestris* [16]], the planthopper *N. lugens* [9], the whitefly *Bemisia tabaci* [17], and the phytophagous mite *Tetranychus evansi* [44]. The conserved and species-specific saliva proteins were identified using BLAST alignment with an E-value cut-off of 10^−5^. Homologous proteins were verified by BlastP searching against the NCBI database, and those that exhibited high sequence similarity and similar annotation were grouped together.

### RNAi, RNAi efficiency, and survival rate

RNAi-mediated gene silencing was conducted as previously described with minor modifications [45]. Briefly, double-strand RNAs (dsRNAs) were synthesized using a T7 high-yield transcription kit (Vazyme) according to the manufacturer’s instructions, with primers containing the T7 RNA polymerase promoter at both ends (Table S1). For dsRNA microinjection, third-instar nymphs were anesthetized with carbon dioxide for 10–15 s. Approximately 700 ng of dsRNA was microinjected into each *P. apterus* abdomen using a FemtoJet microinjection system (Eppendorf). Three days later, salivary glands were dissected from each insect (*n* = 5 for each three replicates) and used for total RNA extraction, followed by cDNA synthesis for RNAi efficiency examination by qRT‒PCR. The survival of dsRNA-treated insects (*n* = 30) was monitored every 24 h.

### qRT‒PCR assay

Total RNA was isolated from whole *P. apterus* or their tissues using RNAiso Plus (Takara) according to the manufacturer’s instructions. First-strand cDNA was synthesized using HiScript QRT super mix (Vazyme). qRT‒PCR was conducted using a CFX96TM real-time PCR detection system (Bio-Rad) with the following conditions: 95 °C for 3 min, followed by 40 cycles at 95 °C for 10 s and 60 °C for 30 s. The relative expression levels of target genes were normalized to the gene encoding ribosomal protein 49 (*rp49*) [45] using the 2^−ΔΔCt^ method (where Ct represents the cycle threshold). Three biological replicates were used for statistical comparison between samples.

### Scanning electron microscopy

Third-instar nymphs were microinjected with dsRNAs targeting *Pyap29161*, *Pyap23512*, *Pyap04329*, *Pyap23515*, *Pyap23508*, *Pyap21548*, *Pyap09885*, and *Pyap23510* and then allowed to feed on red clover seeds. Five days after microinjection, salivary sheaths on the seed surface were attached to a stub, followed by drying in an ion sputter (Ionbeam) under vacuum. After gold sputtering, the samples were observed under a scanning electron microscope (Hitachi) at 3.0 kV.

### Data analysis

Statistical analyses were performed using GraphPad Prism (v8.0). Two-tailed Student’s *t*-tests and log-rank (Mantel‒Cox) tests were used for statistical analysis. Significance levels are indicated as *P* < 0.05 (*), *P* < 0.01 (**), or *P* < 0.001 (***).

## Results

### Identification of P. apterus watery saliva proteins

The results of shotgun LC‒MS/MS revealed 103 peptides under the criterion of an FDR < 0.05 (Table S2). By matching the peptides with the *P. apterus* genomic database, 55 watery saliva proteins were identified, among which 18 proteins exhibited medium or high abundance with unique peptide counts ≥ 2, and 37 proteins had low abundance with only one unique peptide count (Table 1 and S2). In addition, 35 out of 55 (63.6%) watery saliva proteins contained a potential signal peptide, which might facilitate protein secretion from salivary glands to host tissues during feeding (Table 1). Proteomic analysis showed that 19 watery saliva proteins (34.6%) were categorized as digestive enzymes, such as protease, lipase, carboxypeptidase, and carboxypeptidase (Table S2). Eight proteins (14.6%) were assigned to the oxidoreductase category, including glucose dehydrogenase, laccase, and catalase. Ten proteins (18.2%) were non-enzyme proteins, such as transferrin, hexamerin, and odorant-binding protein 10 (OBP10). Notably, 16 (29.1%) watery saliva proteins could not be assigned to any defined functions, among which 11 proteins were specific to *P. apterus* because no homologs were identified in any other genome-available insect species (Table S2).

**Table 1:** Gelling and watery salivary proteins identified by LC‒MS/MS

Presence	Protein ID	Nr annotation	Signal P	Apis^a^	Rpad^a^	Mper^a^	Meup^a^	Dnox^a^	Btab^a^	Nlug^a^	Hhal^a^	Rped^a^	Teva^a^	total
Gelling and watery saliva	Pyap09019	glucose dehydrogenase		Y	Y	Y	Y	Y	Y				Y	7
	Pyap18577	actin, clone 403; hypothetical protein		Y	Y	Y	Y		Y	Y			Y	7
	Pyap20137	glucose dehydrogenase		Y	Y	Y	Y	Y	Y				Y	7
	Pyap17599	F0F1 ATP synthase subunit alpha				Y	Y		Y	Y			Y	5
	Pyap10586	venom serine protease 34-like	Y							Y		Y	Y	3
	Pyap10618	venom serine protease 34-like	Y							Y		Y	Y	3
	Pyap19418	venom serine protease-like	Y							Y		Y	Y	3
	Pyap03843	pancreatic triacylglycerol lipase isoform X2	Y				Y					Y		2
	Pyap12602	carboxypeptidase B-like	Y								Y	Y		2
	Pyap16514	uncharacterized protein LOC109856840					Y					Y		2
	Pyap16515	lipase member I-like					Y					Y		2
	Pyap00120	uncharacterized protein LOC106684787	Y									Y		1
	Pyap00961	perilipin-4-like	Y									Y		1
	Pyap01459	serine protease inhibitor 88Ea-like				Y								1
	Pyap01605	laccase-like	Y									Y		1
	Pyap02134	hexamerin	Y						Y					1
	Pyap13743	lysophospholipid acyltransferase 7	Y									Y		1
	Pyap23510^b^	-	Y									Y		1
	Pyap29089	venom serpin 1; leukocyte elastase inhibitor-like isoform X8	Y			Y								1
	Pyap01349	-	Y											0
	Pyap02377	uncharacterized protein LOC106687672	Y											0
	Pyap02384	uncharacterized protein LOC106683181; unknown secreted protein	Y											0
	Pyap05007	transferrin	Y											0
	Pyap05746	uncharacterized protein LOC106687672	Y											0
	Pyap07146	odorant-binding protein 10												0
	Pyap09083	-	Y											0
	Pyap09227	unknown secreted protein	Y											0
	Pyap11426	hypothetical protein; alpha-glucosidase	Y											0
	Pyap12888	-	Y											0
	Pyap13397	alcohol dehydrogenase class-3												0
	Pyap16563	uncharacterized protein LOC106682594	Y											0
Gelling saliva	Pyap09728	glucose dehydrogenase	Y	Y	Y	Y	Y	Y	Y				Y	7
	Pyap09729	glucose dehydrogenase	Y	Y	Y	Y	Y	Y	Y				Y	7
	Pyap21433	trehalase, partial	Y	Y	Y	Y	Y	Y	Y					6
	Pyap01785	heat shock cognate protein				Y	Y		Y	Y			Y	5
	Pyap11173	aminopeptidase N isoform X3		Y	Y			Y	Y	Y				5
	Pyap17601	F0F1 ATP synthase subunit beta				Y	Y		Y	Y			Y	5
	Pyap19263	apolipophorins	Y	Y	Y	Y			Y				Y	5
	Pyap02756	peroxidase	Y	Y	Y	Y	Y							4
	Pyap03722	uncharacterized protein LOC124461354		Y		Y			Y				Y	4
	Pyap05997	cathepsin L7			Y				Y	Y			Y	4
	Pyap09382	elongation factor 1-alpha				Y			Y	Y			Y	4
	Pyap21105	elongation factor Tu-like				Y			Y	Y			Y	4
	Pyap23124	ubiquitin-40S ribosomal protein S27a			Y	Y			Y				Y	4
	Pyap02664	vitellogenin							Y	Y			Y	3
	Pyap02666	vitellogenin 2, partial	Y						Y	Y			Y	3
	Pyap03092	venom serine protease-like	Y							Y		Y	Y	3
	Pyap05576	venom serine protease-like	Y							Y		Y	Y	3
	Pyap06936	carbonic anhydrase 1-like	Y		Y		Y			Y				3
	Pyap10501	venom carboxylesterase-6-like				Y				Y	Y			3
	Pyap10613	venom serine protease-like	Y							Y		Y	Y	3
	Pyap10616	venom serine protease 34-like	Y							Y		Y	Y	3
	Pyap10620	venom serine protease-like	Y							Y		Y	Y	3
	Pyap02665	-	Y						Y	Y				2
	Pyap02668	vitellogenin	Y						Y	Y				2
	Pyap23530	lipase member H-like					Y					Y		2
	Pyap01412	unkown protein										Y		1
	Pyap01413	unkown protein; probable salivary secreted peptide	Y									Y		1
	Pyap01415	unkown protein; probable salivary secreted peptide	Y									Y		1
	Pyap01416	unkown protein; probable salivary secreted peptide	Y									Y		1
	Pyap01420	unkown protein; probable salivary secreted peptide	Y									Y		1
	Pyap01421	unkown protein; probable salivary secreted peptide	Y									Y		1
	Pyap01612	laccase-like; TATA element modulatory factor isoform X2	Y									Y		1
	Pyap02132	hexamerin	Y						Y					1
	Pyap04080	-	Y									Y		1
	Pyap07020	hypothetical protein	Y									Y		1
	Pyap07242	-											Y	1
	Pyap07407	venom allergen 5-like	Y				Y							1
	Pyap07521	-	Y									Y		1
	Pyap08415	unkown protein; probable salivary secreted peptide										Y		1
	Pyap08711	-	Y									Y		1
	Pyap08713	-	Y									Y		1
	Pyap09250	apolipoprotein D-like	Y										Y	1
	Pyap10416	-	Y						Y					1
	Pyap10820	-	Y										Y	1
	Pyap11032	ankyrin repeat domain-containing protein											Y	1
	Pyap12415	-	Y									Y		1
	Pyap13208	peptidyl-prolyl cis–trans isomerase	Y						Y					1
	Pyap16343	prophenoloxidase							Y					1
	Pyap16366	prophenoloxidase							Y					1
	Pyap16829	-	Y									Y		1
	Pyap19502	rhamnose-binding lectin-like	Y									Y		1
	Pyap20123	-	Y									Y		1
	Pyap20125	-	Y									Y		1
	Pyap21296	protein yellow-like isoform X1	Y									Y		1
	Pyap21548^b^	-	Y									Y		1
	Pyap21549	-	Y									Y		1
	Pyap23500	-	Y									Y		1
	Pyap23505	-	Y									Y		1
	Pyap23508^b^	-	Y									Y		1
	Pyap23509	-	Y									Y		1
	Pyap23511	-										Y		1
	Pyap23512^b^	-	Y									Y		1
	Pyap23514	-	Y									Y		1
	Pyap28697	SHAN3 protein	Y										Y	1
	Pyap01194	pro-resilin-like isoform X4												0
	Pyap02114	neutral ceramidase												0
	Pyap02383	uncharacterized protein LOC106682812 isoform X1	Y											0
	Pyap02925	uncharacterized protein LOC106687669	Y											0
	Pyap02968	secreted venom family 2 protein												0
	Pyap03401	-	Y											0
	Pyap04327	-	Y											0
	Pyap04329^b^	-	Y											0
	Pyap05599	-	Y											0
	Pyap05600	-	Y											0
	Pyap05603	-	Y											0
	Pyap05605	-	Y											0
	Pyap07575	-	Y											0
	Pyap08708	-	Y											0
	Pyap08712	-	Y											0
	Pyap08736	-												0
	Pyap09580	uncharacterized protein LOC106670879; venom protein family 12 protein 1b	Y											0
	Pyap09885^b^	-	Y											0
	Pyap11592	uncharacterized protein LOC106682271 isoform X2	Y											0
	Pyap12262	RNA cytidine acetyltransferase												0
	Pyap12886	uncharacterized protein LOC106679691	Y											0
	Pyap14034	uncharacterized protein LOC106680906	Y											0
	Pyap14628	staufen	Y											0
	Pyap14989	inosine-uridine preferring nucleoside hydrolase												0
	Pyap14990	inosine-uridine preferring nucleoside hydrolase	Y											0
	Pyap15174	programmed cell death protein 10												0
	Pyap15206	lipase 3-like	Y											0
	Pyap16564	uncharacterized protein LOC106682594	Y											0
	Pyap18651	thiol-activated cytolysin family protein, partial	Y											0
	Pyap18652	-	Y											0
	Pyap21254	nucleobindin-2												0
	Pyap21536	-	Y											0
	Pyap21537	-	Y											0
	Pyap21538	-	Y											0
	Pyap21541	-	Y											0
	Pyap21542	-	Y											0
	Pyap21544	-												0
	Pyap21547	-	Y											0
	Pyap21550	-	Y											0
	Pyap22875	salivary secreted cystatin 3 precursor	Y											0
	Pyap23515^b^	-	Y											0
	Pyap23570	-	Y											0
	Pyap23580	-	Y											0
	Pyap24427	30S ribosomal protein S4												0
	Pyap27328	5-dehydro-2-deoxygluconokinase												0
	Pyap29161^b^	-	Y											0
Watery saliva	Pyap06810	glucose dehydrogenase		Y	Y	Y	Y	Y	Y				Y	7
	Pyap15121	aminopeptidase N-like		Y	Y			Y	Y	Y				5
	Pyap03656	uncharacterized protein LOC124461354		Y		Y			Y				Y	4
	Pyap04251	venom s1 protease 12; chymotrypsin-1 isoform X1	Y							Y		Y	Y	3
	Pyap04463	venom serine protease-like								Y		Y	Y	3
	Pyap10606	venom serine protease 34-like	Y							Y		Y	Y	3
	Pyap10607	venom serine protease-like	Y							Y		Y	Y	3
	Pyap10611	venom serine protease-like	Y							Y		Y	Y	3
	Pyap10612	venom serine protease-like	Y							Y		Y	Y	3
	Pyap10624	venom S1 protease 13								Y		Y	Y	3
	Pyap14053	retinoid-inducible serine carboxypeptidase	Y						Y				Y	2
	Pyap23534	lipase	Y				Y					Y		2
	Pyap07018	hypothetical protein	Y									Y		1
	Pyap10261	laccase-like	Y									Y		1
	Pyap26613	30S ribosomal protein S3							Y					1
	Pyap01536	palmitoyl-protein thioesterase 1	Y											0
	Pyap05317	vanin-like protein 2												0
	Pyap09496	catalase isoform X1												0
	Pyap11122	uncharacterized protein LOC106680344	Y											0
	Pyap12535	unknown secreted protein	Y											0
	Pyap17780	shikimate dehydrogenase												0
	Pyap19725	-												0
	Pyap24499	DNA-directed RNA polymerase subunit beta&apos;												0
	Pyap28748	transforming growth factor-beta-induced protein ig-h3	Y											0

^a^*Apis* Acyrthosiphon pisum, *Rpad* Rhopalosiphum padi, *Mper* Myzus persicae, *Meup* Macrosiphum euphorbiae, *Dnox* Diuraphis noxi, *Btab* Bemisia tabaci, *Nlug* Nilaparvata lugens, *Hhal* Halyomorpha halys, *Rped* Riptortus pedestris, *Teva* Tetranychus evansi

^b^The genes were used for RNAi-mediated knockdown

### Identification of P. apterus gelling saliva proteins

The salivary sheath is formed by the secretion of gelling saliva (Fig. 1). A total of 141 proteins were identified in the salivary sheath through shotgun LC‒MS/MS analysis (Table 1 and S3), among which 103 (73.1%) proteins were potentially secretory owing to the presence of a putative signal peptide. Two vitellogenin homologs (vitellogenin 2 and vitellogenin) displayed the highest abundance, with 33 and 30 unique peptide counts, respectively, followed by a zonadhesin-like protein with 29 unique peptide counts. In addition, the gelling saliva contained 38 enzymes, including one isomerase, two acetyltransferases, two ATP synthases, ten oxidoreductases, and 23 digestive enzymes (Table S3). Among the ten oxidoreductases, glucose dehydrogenase was most common, followed by prophenoloxidase and laccase-like proteins (Tables S3). Among the 23 digestive enzymes, 17 (73.9%) were protease and lipases, and two were nucleoside hydrolases. Additionally, the gelling saliva contained 73 proteins with undefined functions, among which 27 proteins were specific to *P. apterus* because no homologs were identified in any other genome-available insect species (Table 1).

### Conserved and species-specific salivary components

**Fig.2:**
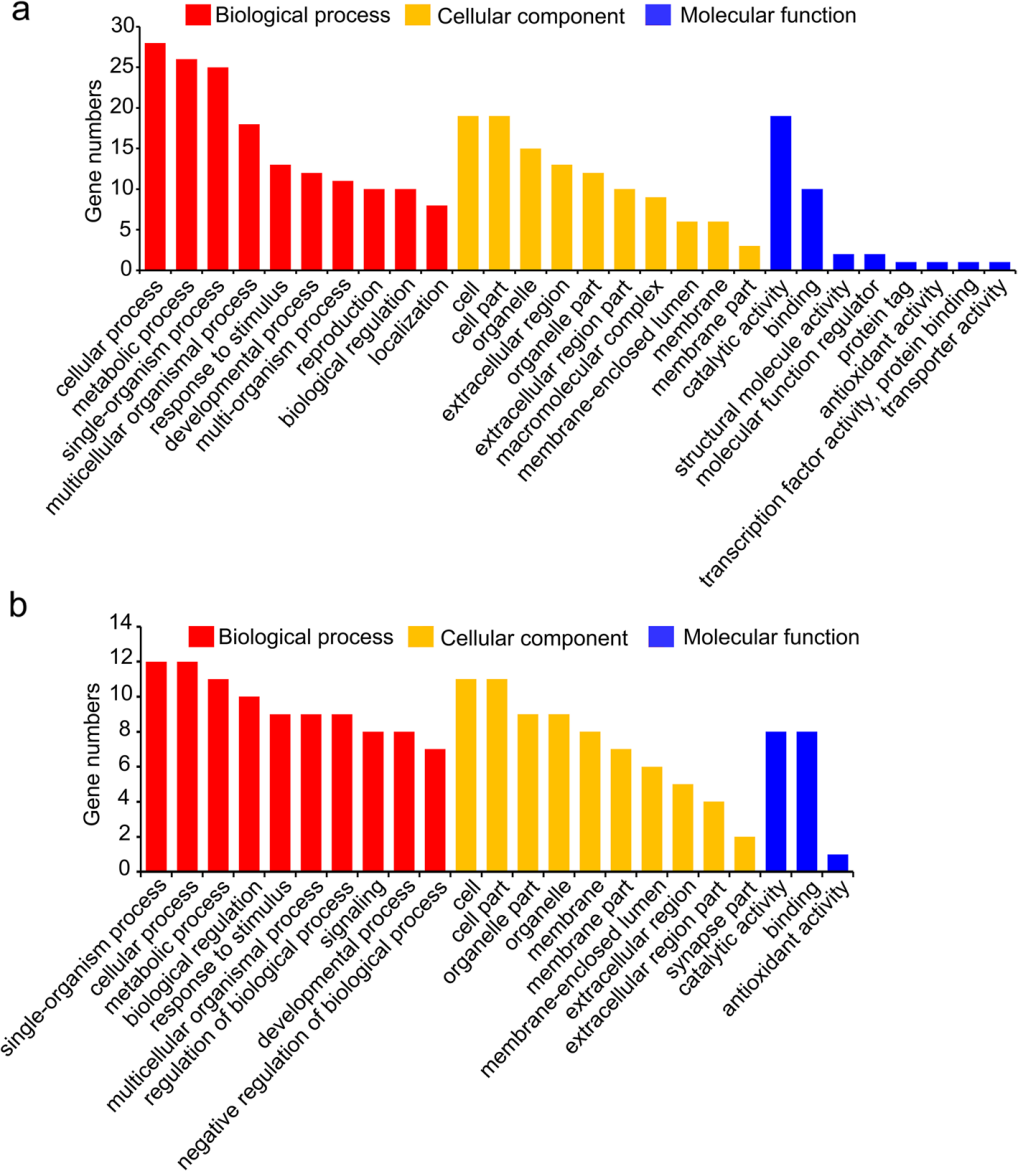
Gene ontology classification of *P. apterus* salivary proteins. **a** Gene ontology classification of the 98 conserved *P. apterus* salivary proteins. **b** Gene ontology classification of the 67 *P. apterus*-specific salivary proteins

In total, 165 *P. apterus* salivary proteins were obtained: 110 gelling-specific proteins, 24 watery-specific proteins, and 31 common proteins in both (Table 1). To provide insights into the functional specificity of *P. apterus* saliva, *P. apterus* saliva proteins were blasted with those derived from ten phytophagous arthropod species. Of the 165 *P. apterus* saliva proteins, 98 (59.4%) had counterparts in the ten other species of arthropods examined (Table 1), such as heat shock cognate proteins, apolipophorins, and enzymes (e.g., protease, aminopeptidase, ATP synthase, trehalase, carbonic anhydrase, and glucose dehydrogenase). Notably, actin was commonly detected in the saliva of all ten arthropods in addition to *P. apterus*. This observation indicates that the common 98 proteins are widely distributed in seed- and phloem-feeding insects. GO enrichment analysis showed that the 98 common salivary proteins were classified into three GO categories at the second level: biological process, cellular component, and molecular function (Fig. 2a and Table S4). The three most enriched GO terms in the ‘biological process’ comprised cellular process (28 proteins), metabolic process (26 proteins), and organic substance metabolic process (25 proteins); the ‘cellular component’ category included cell (19 proteins), cell part (19 proteins), and intracellular part (17 proteins); and the ‘molecular function’ category included catalytic activity (19 proteins), hydrolase activity (11 proteins), and binding (10 proteins). Except for the common 98 proteins, some saliva proteins, such as vitellogenin, apolipoprotein, and hexamerin, were also readily detected across certain phytophagous species. For example, vitellogenin was found to be a component of *P. apterus*, *B. tabaci*, and *N. lugens* saliva, although it was absent in aphid saliva, indicating that it might be conserved in lineage-specific sap-sucking insects.

In addition, 67 out of 165 (40.6%) proteins were only identified in *P. apterus* saliva. GO enrichment analysis showed that: (i) the single-organism process (12 proteins), cellular process (12 proteins), and cellular metabolic process (11 proteins) represented the top three enriched biological processes; (ii) the cell (11 proteins), cell part (11 proteins), and organelle (nine proteins) were the major cellular components, and (iii) the catalytic activity (eight proteins), binding (eight proteins), and antioxidant activity (one protein) were the most enriched molecular functions (Fig. 2b and Table S5).

### Transcriptomic analysis of the P. apterus salivary gland

To fully identify potential salivary proteins, *P. apterus* salivary glands were collected for RNA-seq. After filtering out low-quality reads, adaptor sequences and reads with high levels of unknown bases, a total of 54,340,018 clean reads were obtained from the library, of which over 88.7% reads could be mapped to the *P. apterus* draft genome. Of these, 9,029 (29.88%) genes were potentially expressed in salivary glands with an FPKM > 0 (Table S6), of which 567 proteins contained a predicted signal peptide, but lacked a transmembrane domain, indicating that they might be secreted into saliva by salivary glands (Table S6). Notably, 87 out of 567 genes were commonly detected by LC‒MS/MS and transcriptomic approaches.

### Spatiotemporal expression patterns of P. apterus salivary proteins

**Fig. 3:**
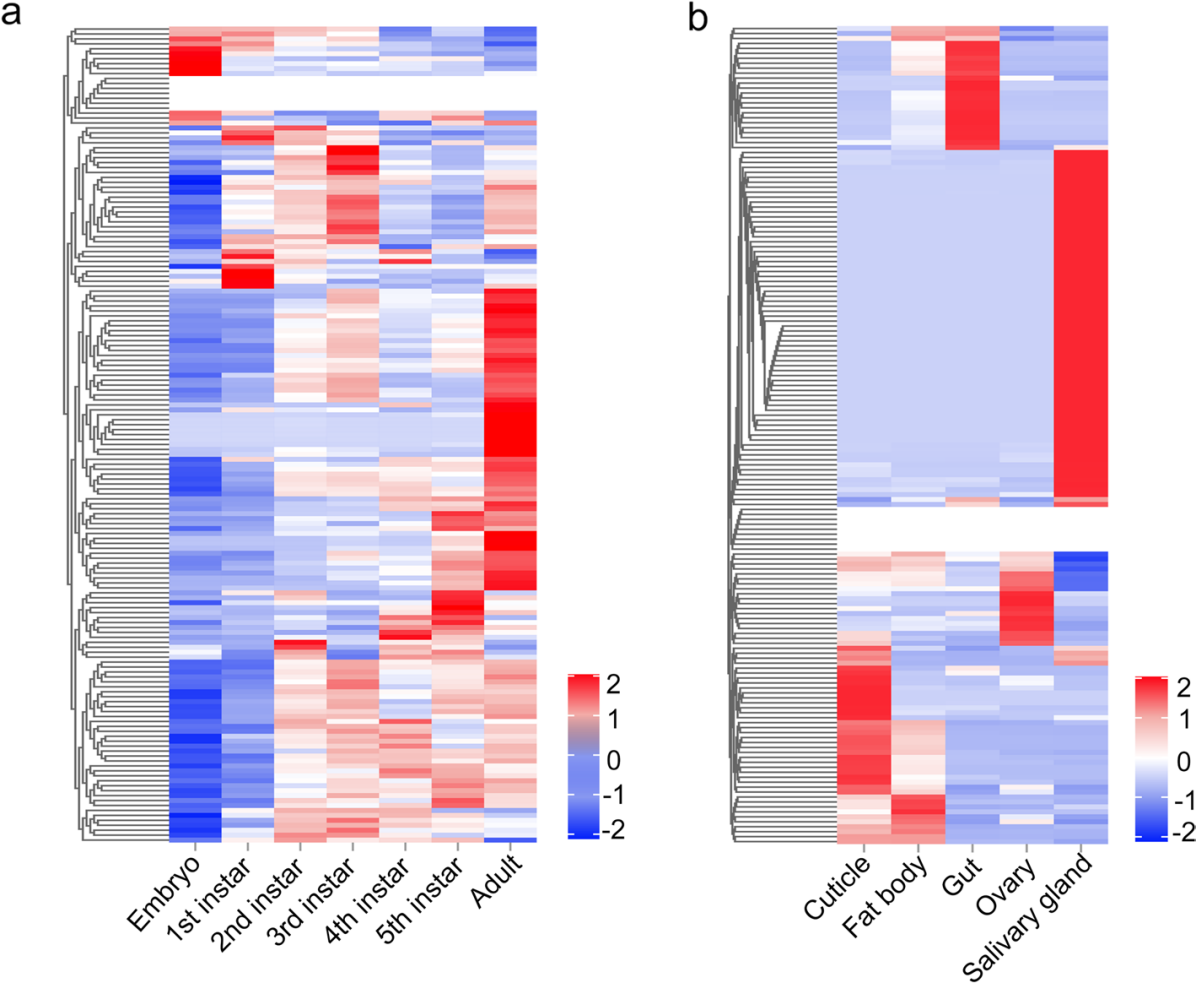
Heatmap depicting the spatiotemporal expression of salivary genes. Different developmental stages of *P. apterus* and different tissues of adults were collected for RNA-seq. Gene expression was evaluated by FPKM. The FPKM of each gene in the same row was *z*-score transformed. **a** The expression of salivary genes across developmental stages. **b** The expression of salivary genes in different tissues of 3-day-old adult females. Color key corresponds to row *z*-score

To investigate the temporal expression patterns of *P. apterus* salivary genes, *P. apterus* at different developmental stages and different body parts of fifth-instar nymphs were collected for RNA-seq, and the gene expression level was quantified using FPKM. This test showed that the majority of salivary genes were stably expressed in nymph and adult stages (Fig. 3a), compared with 7.9% genes that were biased-expressed in eggs. Notably, vitellogenin was exclusively expressed in the adult stage, followed by the high expression of hexamerin and palmitoyl-protein thioesterase 1 (Table S7). The spatial expression patterns indicated that 70 salivary genes were exclusively expressed in the salivary gland (Fig. 3b and Table S8), among which 19 genes encode enzymes such as proteases (13), lipases (2), a glucose dehydrogenase, a carboxypeptidase B-like, a lysophospholipid acyltransferase 7, and a peroxidase. This observation suggests that these genes are important for extra-oral digestion. In addition, 22 genes were highly expressed in the gut, such as those encoding cathepsin L7, inosine-uridine preferring nucleoside hydrolase, and palmitoyl-protein thioesterase 1, suggesting that these are required for internal digestion. Notably, two genes encoding alpha-glucosidase (*Pyap11426*) and lipase (*Pyap23534*) had significantly high transcript levels in both the salivary gland and gut, indicating that they might be important for both oral and internal digestion.

### RNA-mediated knockdown of P. apterus-specific salivary genes

**Fig. 4:**
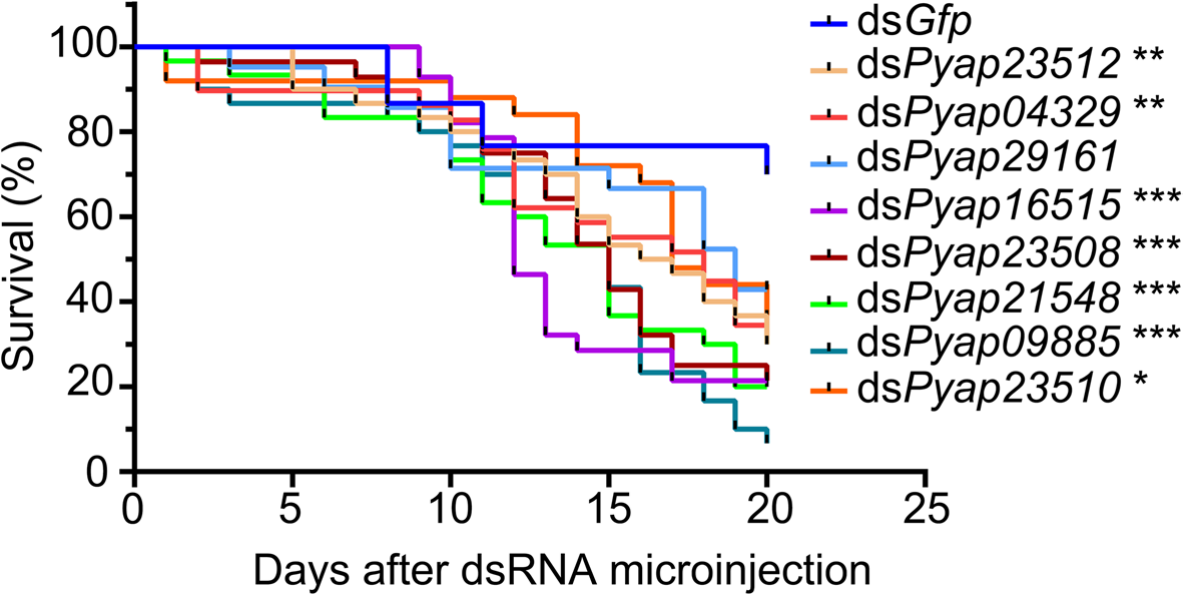
Survival rate of *P. apterus* with gene knockdown. Third-instar nymphs (*n* = 30) were microinjected with dsRNA. The survival of *P. apterus* was monitored every 24 h. Statistical analysis was performed using the log-rank Mantel–Cox test (*P < 0.05, **P < 0.01, and *** *P* < 0.001)

**Fig. 5:**
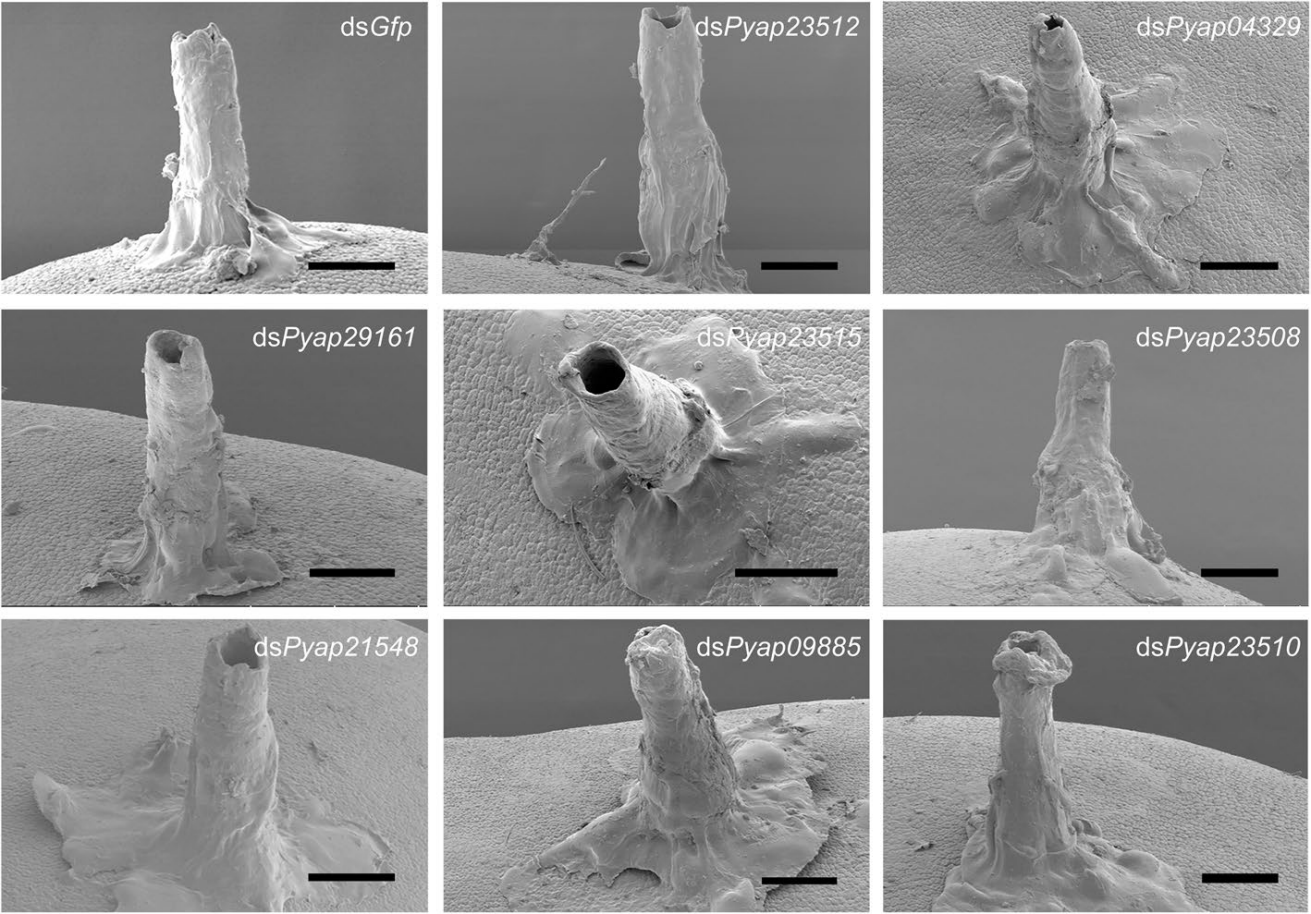
Morphological observations of the salivary sheath secreted by *P. apterus* with gene knockdown. Third-instar *P. apterus* nymphs were injected with dsRNAs and allowed to feed on red clover seeds. Five days after microinjection, the salivary sheaths on the seed surface were observed under a scanning electron microscope. Scale bars: 50 μm

Given that 13 *P. apterus*-specific salivary proteins were assigned with undefined functions (Table 1), we investigated how they contribute to the success of *P. apterus*. To this end, eight genes were randomly selected (*Pyap29161*, *Pyap23512*, *Pyap04329*, *Pyap23515*, *Pyap23508*, *Pyap21548*, *Pyap09885,* and *Pyap23510*) for RNAi-mediated knockdown assays. These 8 genes exhibited stable expression in both nymph and adult stages, with lower expression levels during the embryonic stage (Fig. S1a). Furthermore, the spatial expression patterns indicated that these genes were exclusively expressed in the salivary gland (Fig. S1b). Fourth-instar *P. apterus* nymphs were microinjected with dsRNAs targeting each gene, and the RNAi efficiency was examined by qRT‒PCR three days later. dsRNA treatments significantly reduced the expression of each gene relative to the ds*Gfp* treatment (Fig. S2). Except for *Pyap29161*, knockdown of the remaining genes significantly decreased nymph survival by > 60% (Fig. 4), although it did not impair the deposition of salivary sheaths (Fig. 5). Notably, knockdown of *Pa09885* even resulted in a mortality rate of *P. apterus* as high as 90%. This indicates that undefined salivary proteins might be essential for food ingestion and digestion by *P. apterus*.

## Discussion

The secretion of saliva is an efficient way for insects to digest and absorb nutrients from plant tissues and seed content [46]. Despite extensive studies on the salivary components of phloem-sucking insects [6, 12, 16–18, 24, 28, 46, 47], little is known about the oral secretions of insects that feed on dry seeds with a piercing–sucking mouthpart. In previous studies, to determine the saliva component in several phloem-sucking insects, saliva was collected from artificial food for LC‒MS/MS analysis [6, 17, 44, 48]. Alternatively, salivary glands were dissected for RNA-seq, and potential saliva proteins were predicted based on the presence of a signal peptide but the absence of transmembrane domains [27, 49, 50]. In this study, we identified 165 salivary proteins from the artificial diet via LC‒MS/MS analysis, and 576 potential salivary proteins were predicted from salivary glands via transcriptomic analysis. We noticed that the number of salivary proteins obtained from LC‒MS/MS was less than that obtained from transcriptomic analysis. This discrepancy might be the result of differences in the diet fed to *P. apterus* in the experiments, with insects fed an artificial diet and seeds for LC‒MS/MS and transcriptomic analysis, respectively. Consistent with this observation, previous studies indicated that the saliva composition of *B. tabaci* and *Spodoptera frugiperda* changed along with different host plants [17, 51]. Given that artificial diets were used for saliva collection in most published studies, the accurate composition of insect saliva during host feeding needs further clarification. In addition, we found that 33 out of 165 (20%) *P. apterus* saliva proteins from LC‒MS/MS lacked signal peptides. This indicates that the presence of signal peptides should not be used as the only criterion for screening saliva proteins from the transcriptome.

The firebug *P. apterus* feeds on various seeds that are primarily rich in lipids and proteins via extra-oral digestion, in which the insect repeatedly thrusts its stylet back and forth until several cells have been broken down, enabling it to flush out the seed content with a flow of saliva [30, 52, 53]. For insects performing extra-oral digestion, the secretion of digestive enzymes, such as trypsin and chymotrypsin, is crucial to break down solid contents into liquids, thus increasing food extraction efficiency [54]. In line with this, genes associated with proteolysis and lipid transport were highly expressed in *P. apterus* salivary glands, and numerous proteases and lipases were found in the watery saliva, indicating the importance of watery saliva on extra-oral digestion in *P. apterus*. This observation differs greatly from that in phloem-feeding insects, which rarely secrete proteases during feeding [55, 56]. In addition, a catalase was found in *P. apterus* watery salivary, suggesting that this enzyme is required by *P. apterus* for detoxifying plant-defense compounds, as reported for other hemipterans [5].

For phloem-sucking insects, gelling saliva is continuously secreted during stylet penetration, and then rapidly hardens to protect stylets from physical damage via forming a protective sheath [8, 57]. We noticed that *P. apterus* gelling saliva contained abundant enzymes involved in digestion, including protease, lipase, glucosidase, trehalase, nucleoside hydrolase, carboxypeptidase and aminopeptidase (Table 1). This suggests that gelling saliva is involved in extra-oral digestion during seed feeding in addition to providing a protective sheath. It is well known that phloem-feeding insects encode cell-degrading enzymes, such as amylase, pectinase, cellulose and pectinesterase, to facilitate stylet penetration into plant tissue [5]. Intriguingly, these proteins were not identified in *P. apterus* gelling saliva. A possible explanation for this discrepancy could be the limited phloem sap-sucking behavior of *P. apterus*. In addition, *P. apterus* gelling saliva contained several oxidoreductases, including peroxidase, carboxylesterase, glucose dehydrogenase, prophenoloxidase, and laccase, which were interpreted as a reductive weapon against plant phenols and reactive species [58]. These proteins might be essential for detoxifying secondary metabolites during seed feeding since dry seeds contain abundant phenolic compounds [59]. Oxidoreductases are also commonly identified in the saliva of phloem-feeding insects and use dto detoxify various phenolic compounds produced by stylet penetration [5].

We noticed that carbonic anhydrase was identified in *P. apterus* saliva, which is also present in the saliva of planthoppers, aphids, whiteflies, and leafhoppers [9, 17, 40, 41, 60]. Carbonic anhydrase is a metalloenzyme that catalyzes the conversion of carbon dioxide to bicarbonate ions and protons to mediate pH homeostasis [61]. Previous studies indicated that carbonic anhydrase had an essential role in maintaining alkaline pH for homeostasis and ion transport in the silk gland of the silkworm *Bombyx mori* [62] and in the mosquito gut [63]. However, suppression of carbonic anhydrase gene expression did not change the pH value in either tissue but caused a profoundly lethal effect in the planthopper *N. lugens* [9]. Hence, the detailed function of carbonic anhydrase in insect saliva needs further investigation.

Another interesting finding was that vitellogenin was abundantly detected in the *P. apterus* gelling saliva (Table 1). Vitellogenin is generally considered to be a female-specific nutritious protein vital for oocyte maturation and embryo development in most oviparous vertebrate and invertebrate animals [64]. In recent years, proteomic analysis revealed that vitellogenin is widely distributed in the saliva of piercing–sucking phytophagous arthropods, such as whiteflies, aphids, spider mites, Asian citrus psyllids, and rice planthoppers [6, 9, 44, 48, 65]. The C-terminal polypeptide of vitellogenin in the planthopper *Laodelphax striatellus* acts as an effector that hinders the accumulation of hydrogen peroxide and rice defenses, thereby improving insect feeding performance and survival [65]. In another report, vitellogenin was found to act as a pathogenic transporter to facilitate virus movement from the insect vector into the plant [66]. In addition to vitellogenin, apolipophorin was also identified in the gelling saliva of *P. apterus*, as also found in planthoppers and aphids. Apolipophorin is a protein component of lipoproteins that participates in lipoprotein metabolism, lipid transport, and the immune response in insects [67, 68]. Interestingly, salivary apolipophorin was thought to interfere with plant signaling defense responses [5]. These events suggest that vitellogenin and apolipophorin have important roles in nutrient binding and transporting, and assisting the feeding process.

Odorant-binding proteins (OBPs) are a group of extracellular proteins that are extensively expressed in chemosensory tissues and are known to mediate olfactory transduction by transporting odorant molecules to olfactory receptors [69]. OBP10 was identified in *P. apterus* saliva, in line with multiple other OBPs detected in the salivary glands of various predaceous and hematophagous insects. OBP56a of the blow fly *Phormia regina* saliva solubilizes fatty acids during feeding and subsequently helps to deliver fatty acids to the midgut [70]. The OBP-like protein D7 of a blood-sucking mosquito shows anti-hemostatic and anti-inflammatory action to facilitate blood feeding [71]. In the phytophagous planthopper *N. lugens*, OBP11 assists feeding on rice and acts as an effector that inhibits plant defense [72]. The secretion of OBP10 in *P. apterus* saliva suggests that it contributes to *P. apterus* feeding on animal matter and live plants, but its precise function in this species remains unknown.

Many studies have indicated significant variations in saliva composition among insect species, and the presence of species-specific salivary proteins might be essential for feeding. For example, the presence of Ca^2+^-binding proteins in the saliva of the Sternorrhyncha could aid their phloem feeding by reducing the occlusion of sieve-tube elements, which would occur as an induced defense response in plants [5]. To better understand the function of species-specific salivary proteins that are exclusively expressed in the salivary gland, we used RNAi to decrease the expression level of eight genes encoding gelling salivary proteins. Silencing these genes led to a significant reduction in *P. apterus* survival but did not alter the deposition of salivary sheaths. This event strongly indicates that gelling salivary proteins not only serve as a stylet sheath but also function to facilitate host feeding. It was found that these eight genes are only present in *P. apterus* and *Riptortus pedestris,* which can also feed on dry seeds (Table 1). They are almost absent in phloem- and blood-feeding insects, suggesting that the presence of these proteins may aid truebugs in seed feeding. However, the scarcity of research on salivary components in other insects that feed on dry seeds poses a challenge in determining whether these proteins are commonly found in seed-feeding insects.

Overall, our study identified and characterized the salivary proteins of *P. apterus* using proteomic and transcriptomic approaches. The results derived from our study will be helpful for understanding the feeding process of sap-sucking and seed-feeding insects.

## Supplementary Information

**Additional file 1: Fig. S1. **HeatmapHeat map illustrating the spatiotemporal expression of the 8 salivary genes used for RNAi. Different developmental stages of *P. apterus* and different tissues of adults were collected for RNA-seq. Gene expression was evaluated by FPKM. **a** The expression of 8 salivary genes across developmental stages. **b** The expression of 8 salivary genes in different tissues of 3-day-old adult females. Color key corresponds to row *z-score.*

**Additional file 2: Fig. S2. **Examination of RNAi efficiency by qRT–PCR. Third-instar nymphs were collected for dsRNA microinjection. Three days later, salivary glands were dissected from insects (n = 5 for each three replicates) and used for total RNA extraction. The relative expression of each gene was normalized to the expression of *rp49*. Data are presented as the mean± SEM. Two-tailed unpaired Student’s *t*-test was used for the statistical analysis (*P< 0.05, and **P < 0.01).

**Additional file 3: Table S1. **Main primers used in this study.

**Additional file 4: Table S2. **Watery salivary proteins of *P. apterus*.

**Additional file 5: Table S3. **Gelling salivary proteins of *P. apterus*.

**Additional file 6: Table S4. **Gene ontology classification of the conserved *P. apterus* salivary proteins.

**Additional file 7: Table . S5.** Gene ontology classification of *P. apterus*-specific salivary proteins.

**Additional file 8: Table S6. **Gene expression level in salivary glands detected by transcriptomics.

**Additional file 9: Table S7. **Temporal expression of *P. apterus* salivary genes.

**Additional file 10: Table S8. **Spatial expression of *P. apterus* salivary genes.

## Data Availability

Illumina sequencing data have been submitted to the National Center for Biotechnology Information (NCBI) Sequence Read Archive (SRA) under Bio- Project PRJNA954282. The LC‒MS/MS proteomics data were deposited to the ProteomeXchange Consortium (GenBank project accession: PXD040558).
