# Supplementary figures and images for: LC‒MS/MS and transcriptome analyses reveal saliva components of the seed-feeding truebug Pyrrhocoris apterus
Source: Crop Health. 2023 Dec 15;1(1):20. doi: 10.1007/s44297-023-00021-w (PMC12825922; doi:10.1007/s44297-023-00021-w)

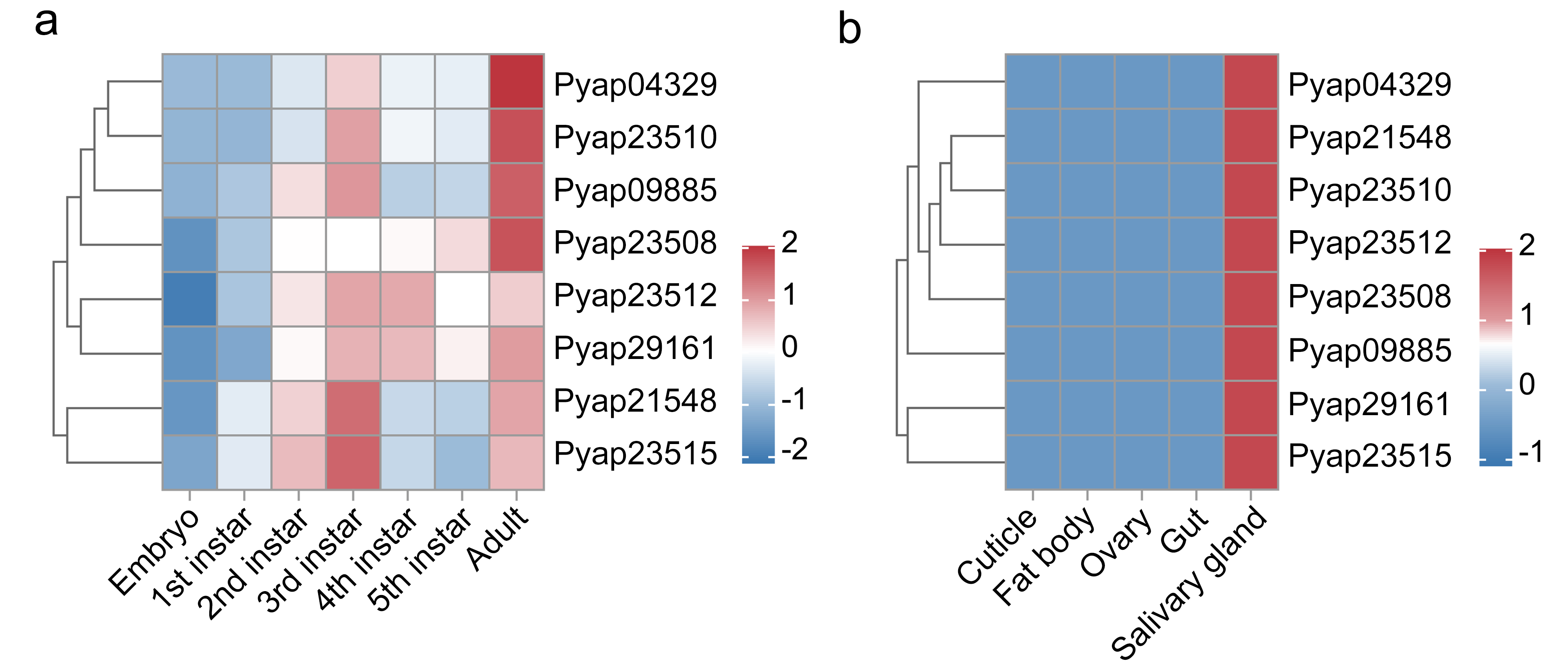

Supplement: Supplementary file 1 — Additional file 1: Fig. S1. HeatmapHeat map illustrating the spatiotemporal expression of the 8 salivary genes used for RNAi. Different developmental stages of P. apterus and different tissues of adults were collected for RNA-seq. Gene expression was evaluated by FPKM. a The expression of 8 salivary genes across developmental stages. b The expression of 8 salivary genes in different tissues of 3-day-old adult females. Color key corresponds to row z-score. [file 44297_2023_21_MOESM1_ESM.tif]

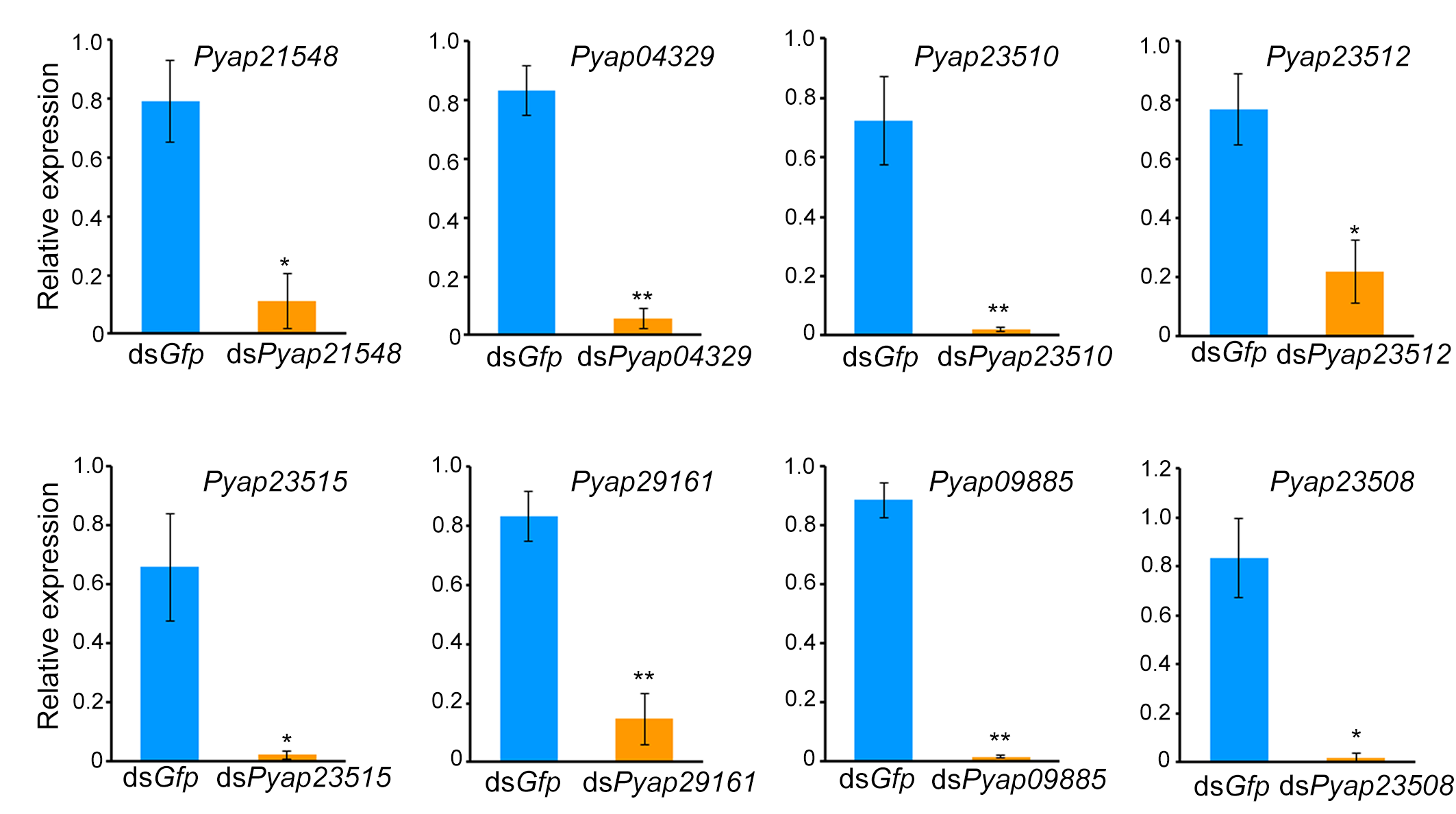

Supplement: Supplementary file 2 — Additional file 2: Fig. S2. Examination of RNAi efficiency by qRT–PCR. Third-instar nymphs were collected for dsRNA microinjection. Three days later, salivary glands were dissected from insects (n = 5 for each three replicates) and used for total RNA extraction. The relative expression of each gene was normalized to the expression of rp49. Data are presented as the mean± SEM. Two-tailed unpaired Student’s t-test was used for the statistical analysis (*P< 0.05, and **P < 0.01). [file 44297_2023_21_MOESM2_ESM.tif]
